# Predicting Attitudes toward Press- and Speech Freedom across the U.S.A.: A Test of Climato-Economic, Parasite Stress, and Life History Theories

**DOI:** 10.1371/journal.pone.0125241

**Published:** 2015-06-01

**Authors:** Jinguang Zhang, Scott A. Reid, Jing Xu

**Affiliations:** 1 Department of Communicology, University of Hawai‘i, Mānoa, Honolulu, HI, United States of America; 2 Department of Communication, University of California Santa Barbara, Santa Barbara, CA, United States of America; 3 Annenberg School of Communication, University of Southern California, Los Angeles, CA, United States of America; University G. d'Annunzio, ITALY

## Abstract

National surveys reveal notable individual differences in U.S. citizens’ attitudes toward freedom of expression, including freedom of the press and speech. Recent theoretical developments and empirical findings suggest that ecological factors impact censorship attitudes in addition to individual difference variables (e.g., education, conservatism), but no research has compared the explanatory power of prominent ecological theories. This study tested climato-economic, parasite stress, and life history theories using four measures of attitudes toward censoring the press and offensive speech obtained from two national surveys in the U.S.A. Neither climate demands nor its interaction with state wealth—two key variables for climato-economic theory—predicted any of the four outcome measures. Interstate parasite stress significantly predicted two, with a marginally significant effect on the third, but the effects became non-significant when the analyses were stratified for race (as a control for extrinsic risks). Teenage birth rates (a proxy of human life history) significantly predicted attitudes toward press freedom during wartime, but the effect was the opposite of what life history theory predicted. While none of the three theories provided a fully successful explanation of individual differences in attitudes toward freedom of expression, parasite stress and life history theories do show potentials. Future research should continue examining the impact of these ecological factors on human psychology by further specifying the mechanisms and developing better measures for those theories.

## Introduction

Democracy requires the freedom to publicly express one’s views without fear of retribution. Such freedom is a social good that provides checks and balances on power and enables social and technological innovation. Despite these benefits, people tolerate dissent to different degrees. Women [1], the elderly [2], the less educated [3], and people who are more authoritarian, politically conservative, and religious [4–6] are more likely to endorse censoring the press and speech (i.e., they are less tolerant of dissent).

Research has begun to move from examining individual-level correlates of censorship attitudes to examining wider ecological impacts. For example, climato-economic theory [7] and parasite stress theory [8], two prominent ecological theories, have been invoked to explain between-country differences in institutional repression of media freedom [8,9]. The former theory posits an interaction effect of local climate demands and wealth, while the latter focuses on the effect of local levels of parasite stress. Yet another important ecological theory, life history theory [10], has been used to explain individual and cross-cultural differences in political orientation [11] and religiosity [12], both of which predict censorship attitudes (see above). No research, however, has directly compared the predictive power of these three theoretical explanations. The present study filled this void in the literature by testing the predictions of climato-economic, parasite stress, and life history theories on U.S. citizens’ attitudes toward press- and speech freedom, two core components of freedom of expression.

### A Climato-Economic Theory Explanation

Climato-economic theory [7] proposes a causal link from regional differences in climate demands and financial resources to cross-cultural patterns of human values. The theory starts with the observation that a temperate climate (e.g., a yearly average of approximately 72°F) provides high thermal comfort, abundant nutritional resources, and healthy habitats. As the climate deviates from this temperate ideal, living conditions become more stressful and survival costs increase. The lack of financial resources makes it difficult for residents living in more demanding climates to compensate for high survival costs. Thus, residents of these regions (e.g., Kyrgyzstan) prioritize ingroup cooperation to mitigate against the survival threats. Over time, they become highly collectivistic, develop autocratic leadership, and form centralized social structures. A consequence is heightened governmental control over the press [9].

In contrast, people living in wealthy regions with harsh climates experience less stress because wealth provisions against threats to survival. Residents of such regions (e.g., Finland) emphasize self-actualization and see individual effort as the primary means to achieve it. Over time, they become highly individualistic, develop democratic leadership, and form decentralized social structures. A consequence is lessened governmental control over the press [9].

Climato-economic theory thus predicts that increased climate demands will be associated with more freedom of expression in wealthy regions but less freedom of expression in poorer regions. Van de Vliert [9] confirmed this prediction using data on governmental repression of the press across 175 countries. However, given that the theory specifies the effects of climate and wealth on human values, a more direct test would be to model citizen’s attitudes toward free expression, rather than the institutions assumed to emerge from those attitudes.

### A Parasite-Stress Theory Explanation

Parasites are a major cause of human morbidity and mortality that have created strong selection pressures that favor adaptations for disease avoidance. While the physiological immune system provides a defense against pathogens that have entered the body, parasite stress theory [8] posits that a complementary “behavioral immune system” [13] has evolved to decrease the likelihood of contact with pathogens. Because pathogens and human hosts engage in co-evolutionary races, people develop a degree of immunity to local but not non-local pathogenic diseases. Members of outgroups thus represent a pathogenic threat because they are more likely than ingroup members to host novel diseases.

According to parasite stress theory, political conservatism facilitates disease avoidance because it promotes outgroup avoidance (i.e., xenophobia; [14]) and strong ingroup ties (i.e., ethnocentrism; [15]). Conservatism also emphasizes resistance to change [16], thus helping preserve norms. Confirming these conceptual analyses, research shows that cross-cultural parasite stress positively predicts authoritarianism [17,18] and valuation of obedience [19], but negatively predicts openness to new experiences [20]. At an individual level, disgust sensitivity—an emotional component of behavioral immunity—positively predicts social and political conservatism [21], and increases in perceived vulnerability to disease and disease primes lead to stronger conformist attitudes [22].

Freedom of expression encourages dissent, thereby threatening social convention. Freedom of expression can also breed intense interpersonal and intergroup conflict (e.g., through offensive speech) that threatens social stability. As such, free expression counters the logic of the behavioral defense against parasites (which favors social stability and convention), and should be devalued when local parasite stress is high. Thus, if parasite stress theory is correct, regional parasite stress will positively predict support for restricting press- and speech freedom.

### A Life-History Theory Explanation

Life history theory [10] describes how humans (and other animal species) strategically allocate their finite time, energy, and material resources between different fitness components in a given ecology. Individuals who prioritize future reproduction, offspring quality, and parental effort (among other long-term goals) are said to adopt a “slow” life-history strategy (LHS). Those who emphasize present reproduction, offspring quantity, and mating effort (among other short-term goals) are said to adopt a “fast” LHS.

The adoption of a slow or fast LHS is individually variable [23] and ecologically sensitive [24]. Life history theory posits that individuals living in secure and stable environments are more likely to become slow LH strategists, and this is because these individuals have a large chance of surviving into the future to reap the benefits accrued through investments in somatic development and parental effort. In contrast, individuals living in harsh and unstable environments are likely to become fast LH strategists. This is because they are less likely to survive into the future to reap long-term benefits, and early and immediate reproduction yields a larger payoff for them. Research supports the application of life history theory to the study of human psychology and behavior, such as decisions in financial investment and timing of reproductive effort [25,26].

Life history theory may predict censorship attitudes through political ideology. Thornhill and Fincher [11] hypothesized that conservatism is developmentally linked to a secure attachment style, which is the result of growing up in a secure environment and thus the adoption of a slow LHS. Consistent with this hypothesis, they found that fewer childhood stressors (e.g., absence of a parent) were associated with higher levels of self-report political conservatism. If this is true, and given that conservatives are more likely to endorse censorship, it can be expected that a slower LHS will correlate with stronger support for restricting press- and speech freedom.

LH strategies may also predict censorship attitudes through moral concerns. According to Gladden, Welch, Figueredo, and Jacobs [27], because a slow LHS is associated with a stable environment, slow LH strategists will be more motivated than fast LH strategists to create and enforce a stable environment by moralizing social interactions. Because freedom of expression can disrupt social stability and cause harm to others, it can also be expected that a slower LH strategy will correlate with stronger censorship attitudes.

### The Present Research

In this research, we tested the three ecological explanations using four measures of attitudes toward freedom of expression extracted from two U.S. national surveys—the State of the First Amendment Surveys (firstamendmentcenter.org) and the General Social Survey (norc.org). Tests 1 and 2 were on attitudes toward press freedom, and Tests 3 and 4 were on attitudes toward speech freedom. We first evaluated each theoretical explanation independently and then performed competitive tests. Table 1 summarizes the three theories and their predictions.

**Table 1:** Overview of theoretical accounts and their predictions.

Theories	Predictions
Life-history	Slower LH → stronger support for restricting press freedom
	Slower LH → stronger support for restricting speech freedom
Parasite-stress	Higher parasite stress → stronger support for restricting press freedom
	Higher parasite stress → stronger support for restricting speech freedom
Climato-economic	Higher climate demands → stronger support for restricting press- and speech freedom among poorer states

## Materials and Methods

Table 2 summarizes the four outcome measures and their source.

**Table 2:** Purpose and example items of the four tests as well as the surveys from which the items were drawn.

	Purpose	Example Items	Item Source
Test1	Attitudes toward press freedom in general	“Overall, do you think the press in America has too much freedom to do what it wants?”	2005 and 2006 State of the First Amendment Surveys
Test 2	Attitudes toward wartime press freedom	“Newspapers should be allowed to freely criticize the U.S. military about its strategy and performance.”	2006 State of the First Amendment Survey
Test 3	Attitudes toward speech freedom	“Musicians should be allowed to sing songs with lyrics that others might find offensive.”	2005 and 2006 State of the First Amendment Surveys
Test 4	Attitudes toward speech freedom	Should a person who is against churches and religion be allowed to make a speech in your community?	2012 General Social Survey

### Test 1: Attitudes toward Press Freedom in General

A single-item measure of attitudes toward press freedom was obtained from the 2005 and 2006 State of the First Amendment Surveys: “Overall, do you think the press in America has too much freedom to do what it wants, too little freedom to do what it wants, or is the amount of freedom the press has about right?” The item was re-coded such that larger values indicate stronger endorsement of restricting press freedom (1 *too little freedom*, 2 *about right*, 3 *too much freedom*).

We used these two surveys because respondents’ residence states were recorded. The entire sample (*N* = 2,005, *Median*_age_ = 54; 53% female) had 80.9 percent non-Hispanics Whites, 7.2 percent African Americans, 4.0 percent Hispanics, and 1.7 percent Asians; the rest were categorized as “bi-racial” and “other.” Three states had fewer than 10 respondents, including Delaware (*n* = 6), Vermont (*n* = 6), and Wyoming (*n* = 2). The sample size for other states ranged from 11 to 186. In our analyses, we will test the three explanations with all states as well as states that had more than 10 respondents.

### Test 2: Attitudes toward Wartime Press Freedom

In the 2006 State of the First Amendment Survey, respondents (*N* = 1,002; *Median*_age_ = 51; 54% female) were asked how much they agreed with each of the following statements: (1) “Newspapers should be allowed to freely criticize the U.S. military about its strategy and performance,” (2) “Newspapers should honor government requests to withhold publishing information that might hurt efforts to win the war on terrorism (reverse coded),” and (3) “Even during wartime, the press should be allowed to publish stories that criticize the actions of the government” (1 *strongly agree*, 4 *strongly disagree*). These items formed a reliable index of attitudes toward wartime press freedom (α = .71). Larger values indicate stronger endorsement of restricting press freedom for national interest.

In this survey, 79.1 percent were non-Hispanic Whites, with 7.5 percent African Americans, 4.5 percent Hispanics, and 1.8 percent Asians; the rest were categorized as “bi-racial.” Thirteen states had fewer than 10 respondents, including Delaware (*n* = 3), Idaho (*n* = 6), Michigan (*n* = 9), Nebraska (*n* = 6), Nevada (*n* = 4), New Hampshire (*n* = 8), North Dakota (*n* = 6), Rhode Island (*n* = 4), South Carolina (*n* = 5), South Dakota (*n* = 5), Vermont (*n* = 9), West Virginia (*n* = 9), and Wyoming (*n* = 2). The sample size for the other states ranged from 11 to 95. We deal with states with relatively fewer respondents in our analyses as above.

### Test 3: Attitudes toward Speech Freedom

In the 2005 and 2006 State of the First Amendment surveys, respondents were also asked how much they agreed with the following statements: (1) “Musicians should be allowed to sing songs with lyrics that others might find offensive”; (2) “People should be allowed to say things in public that might be offensive to racial groups”; and (3) “People should be allowed to say things in public that might be offensive to religious groups” (1 *strongly agree*, *4 strongly disagree*). These items formed a reliable index of attitudes toward speech freedom (α = .79). Larger values indicate stronger endorsement of restricting speech freedom.

### Test 4: Attitudes toward Speech Freedom—A Replication

As a replication of Test 3, we obtained six items on attitudes toward speech freedom from the 2012 General Social Survey. The first item states that “there are always some people whose ideas are considered bad or dangerous by other people. For instance, somebody who is against churches and religion. If such a person wanted to make a speech in your city/town/community against churches and religion, should he be allowed to speak or not” (1 *yes*, *allowed to speak*, 2 *not allowed*). For the remaining questions, the “person” was described as someone who believes that blacks are genetically inferior, is a communist, advocates doing away with elections and letting the military run the country, admits that he is a homosexual, and someone who is a Muslim clergyman who preaches hatred of the U.S. We summed the values across the six items to form a continuous measure of attitudes toward speech freedom (α = .82). Larger values indicate stronger endorsement of speech censorship.

In this survey, respondents (*N* = 4,819; *Median*_age_ = 49; 56% female) were classified into nine regions where the interviews were conducted: New England (*n* = 225), Middle Atlantic (*n* = 596), East North Central (*n* = 831), West North Central (*n* = 301), South Atlantic (*n* = 1041), East South Central (*n* = 289), West South Central (*n* = 480), Mountain (*n* = 364), and Pacific (*n* = 692). There were 76.8 percent Whites and 15 percent African Americans, with the remainder categorized as “other.”

### Measures of Climato-Economic Theory Variables

#### Climate demands

To test climato-economic theory, we first created an index of climate demands for the 48 states following Van de Vliert [7]. For each state, we determined the lowest and highest temperatures (in °F) in the coldest and hottest months that were recorded till 2010 (census.gov). We then calculated the difference between 72°F (i.e., the temperate ideal) and each of the four temperatures before summing the absolute values of the differences.

For example, the lowest temperature for Alabama was 3°F (recorded in January), and the highest temperature in that same month was 84°F. The highest temperature for Alabama was 105°F (recorded in August) and the lowest temperature in that month was 59°F. The climate demand for Alabama thus equaled |72–3| + |72–84| + |72–105| + |72–59| = 127. *Z*-scores were used for analyses, with larger values indicating greater climate demands.

To validate our computation, we calculated the climate-demand scores for all 50 states, and correlated them with Vandello and Cohen’s [28] measure of U.S. collectivism. Our coefficient (*r* = -.73) is almost identical to that reported by Van de Vliert ([2]; *r* = -.72).

#### Wealth

Following previous research [29], we obtained three indices of state economic development: (1) median household income, (2) per capita income, and (3) gross state product per capita (census.gov). For each state, we calculated a mean score of the indices across the years 2005 and 2006 and then standardized the scores. After standardization, the three indices formed a reliable measure of state wealth (α = .91), with larger values indicating greater wealth.

### Measures of Interstate Parasite Stress

We used two measures of interstate parasite stress. The first measure was obtained from Fincher and Thornhill [30] (the “F&T measure”), which is based on records of 47 categories of infectious diseases (e.g., AIDS, Hepatitis, Typhoid fever) from 1993 to 2007. This measure has been used in earlier tests of parasite stress theory in the context of the U.S.A. [29,30].

Recently, Hackman and Hruschka [12] argued that the F&T measure is confounded with two LH variables: 1) sexual strategy and 2) extrinsic mortality rates. Faster LH strategists are more sexually active [31] and thus more likely to contract sexually transmitted diseases (STDs). Hackman and Hruscka showed that, once the STD rates—in particular the rates of Chlamydia and Gonorrhea (C&G rates)—were removed from the F&T measure, the F&T measure no longer predicted variables of interest (e.g., religiosity, strength of family ties; [30]).

At the same time, higher mortality rates due to extrinsic risks (e.g., accidents) cause the adoption of a faster LHS [32]. Given that African Americans have higher extrinsic mortality rates than non-Hispanic Whites in the U.S. [33], Hackman and Hruscka showed that, once they stratified the C&G rates and outcome variables (e.g., religiosity) into African Americans versus Whites (thus controlling for extrinsic mortality rates), the C&G rates no longer predicted the outcome variables within each race category. Because the non-stratified C&G rates strongly correlate with the F&T measure (*r* = .95, *N* = 50; [12]) and, once stratified, control for extrinsic mortality rates (i.e., an important LH variable), stratified C&G rates have since been used for competitive tests of parasite stress and life history theories [34].

Following Hackman and Hruschka [12], we similarly obtained the non-stratified C&G rates (“C&G Total”) and the rates for non-Hispanic Whites (“C&G White”) from Centers for Disease Control and Prevention (cdc.gov). C&G White was used as a second measure of interstate parasite stress in this research to analyze the subsamples of non-Hispanic Whites (cf. [12,35]). We did not compute the C&G rates for African Americans because the two surveys that we used for this research had too few African American respondents (about seven to 15 percent of the samples) to allow for meaningful analyses.

Compared to the use of the F&T measure, the use of C&G rates offered a more conservative test of parasite stress theory, because STDs are only one type of the infectious diseases that have impacted human evolution [35]. For this reason, and because C&G Total also strongly correlated with the F&T measure in this research, *r* = .98, *N* = 48 (excluding Alaska and Hawaii), which rendered the two measures indistinguishable, we used the F&T measure instead of C&G Total to analyze the full sample as a more standard test of parasite stress theory. We also performed the tests using C&G Total, which yielded identical findings to those using the F&T measure. The findings of C&G Total were presented in Table E in S1 File. For both the F&T measure and C&G White, higher values indicated stronger parasite stress in a state.

### Life History Variables

To test life history theory, we obtained measures of teenage birth rates for all race categories (“Teen Birth Total”) and those for non-Hispanic Whites (“Teen Birth White”) (www.guttmacher.org/pubs/USTPtrends.pdf). Hackman and Hruschka [11] showed that teen birth rates, stratified by race or not, reliably predicted religiosity and strength of family ties across U.S. states (but see [34] for several null findings and [35] for a theoretical critique). We similarly used both measures in our tests to compare their effects, with higher values indicating a faster LHS.

### Measures of Individual-Level Control Variables

In both the First Amendment and General Social Surveys, respondents provided information on their gender (1 *male*, 2 *female*) and education level (First Amendment Survey: 1 *grade school or less*, 7 *post-graduate*; General Social Survey: 0 *left high school*, 4 *graduate degree*). Previous research showed that women and less educated people are more likely to endorse censorship ([1,3]), and we thus included them as individual-level covariates. In all tests, gender was dummy-coded (0 = male) and education level was grand-mean centered.

## Results

Data for all tests are available in S1-4 Datasets. Descriptive statistics, including the intercorrelations among all variables, are reported in Table A-E in S1 File.

We used full samples when testing climato-economic theory, and used both the full- and subsamples of non-Hispanic Whites to test parasite stress and life history theories. Because there was no evidence for between-state variances in the outcome measure for Test 1 (i.e., general censorship attitudes), χ^2^ = 1.41, *p* = .24, the effects of ecological variables (i.e., climate demands, state wealth, parasite stress, and teen birth rates) were not assessed. Instead, OLS regressions were used to examine the impact of Level-1 predictor variables (i.e., participants’ education level and gender) on the outcome measure.

We performed multilevel modeling with maximum likelihood estimation for Tests 2 and 3 and restricted maximum likelihood estimation for Test 4; this is because the number of Level-2 units in Test 4 was small (i.e., nine regions). Multilevel modeling was implemented with SPSS version 18. Proportion reduction in variance (PRV; [36]), which equals Variance_no predictor_—Variance_predictor_)/Variance_no predictor_, was used as an effect-size estimate for multilevel models.

### Tests of the Climato-Economic Explanation

Climato-economic theory predicted that climate demands will positively predict censorship attitudes among poorer states but negatively predict censorship attitudes among wealthier states. To test this prediction, we used climate demands, state wealth, and the interaction term of the two as Level-2 predictor variables while controlling for respondents’ gender and education level. Results are summarized in Table 3. There was evidence that state wealth negatively predicted endorsement of censoring the press during wartime (Test 2) and some evidence that it predicted endorsement of censoring hate speech (Test 3). However, there was no evidence for the predicted two-way interaction between climate demands and wealth in any of the four tests. Climato-economic theory was not supported.

**Table 3:** Test of climato-economic theory.

		Press freedom in general (Test 1)	Wartime press freedom (Test 2)	Speech freedom (Test 3)	Speech freedom(Test 4)
Climate demands	*b*		-.02	.003	-.16
	*t*(df)		< 1	< 1	-1.34 (5.52)
	*p*				.23
	Effect size				PRV = 15%
State wealth	*b*		-.11	-.06	.01
	*t*(df)		2.72 (56.60)	-1.87 (48.3)	< 1
	*p*		.009	.068	
	Effect size		PRV = 42%	PRV = 54%	
Climate × wealth	*b*		.03	.01	.19
	*t*(df)		< 1	< 1	< 1
	*p*				
	Effect size				
Education	*b*	-.06	-.14	-.15	-.45
	*t*(df)	-6.35 (1920)	-7.68 (987.5)	-10.5 (1978.2)	-16.4 (3137.1)
	*p*	< .001	< .001	< .001	< .001
	Effect size	β = .14	PRV = 11%	PRV = 55%	PRV = 14%
Gender (male = 0)	*b*	.10	.07	.27	.20
	*t*(df)	3.24	1.19 (990.0)	6.13 (1977.7)	2.95
	*p*	.001	.24	< .001	.003
	Effect size	β = .07	PRV = 9%	PRV = 46%	PRV = 9%

Note. PRV = proportional reduction in variance.

### Tests of the Parasite Stress Explanation

Parasite stress theory predicted that both the F&T measure of interstate parasite stress (i.e., the non-stratified, overall parasite stress) and C&G White (i.e., rates of Chlamydia and Gonorrhea for non-Hispanic Whites) will positively predict support for restricting press- and speech freedom. To test this prediction, we ran separate multilevel models with the F&T measure and C&G White as the respective Level-2 predictor variables. Respondents’ gender and education level were covariates. Results are summarized in Table 4.

**Table 4:** Tests of parasite stress theory.

		Press freedom in general (Test 1)		Wartime press freedom (Test 2)		Speech freedom (Test 3)		Speech freedom (Test 4)	
		State totals	White	State totals	White	State totals	White	State totals	White
Parasite stress	*b*			.10	.06	.08	.003	-.16	-.01
	*t*(df)			2.26 (58.6)	1.71 (39.1)	2.38 (81.9)	< 1	2.10 (9.12)	< 1
	*p*			.027	.095	.020		.065	
	Effect size			PRV = 29%	23%	PRV = 35%		PRV = 36%	
Education	*b*	-.06	-.07	-.14	-.17	-.15	-.14	-.45	-.44
	*t*(df)	-6.35 (1920)	-6.39 (1560)	-7.87 (988.4)	-8.22 (787.8)	-10.6 (1959.7)	-9.15 (1598.4)	-16.4 (3142.0)	-14.5 (2410.7)
	*p*	< .001	< .001	< .001	< .001	< .001	< .001	< .001	< .001
	Effect size	β = .14	.16	PRV = 19%	34%	PRV = 52%	42%	PRV = 13%	12%
Gender (male = 0)	*b*	.10	.08	.07	.02	.27	.25	.19	.27
	*t*(df)	3.24	2.57	1.17 (988.4)	< 1	6.17 (1977.4)	5.20 (1607.1)	2.94 (3137.3)	3.58 (2407.3)
	*p*	.001	.010	.24		< .001	< .001	.003	< .001
	Effect size	β = .07	.06	PRV = 5%		PRV = 29%	24%	PRV = 11%	12%

Notes. State totals = non-stratified analyses based on Fincher & Thornhill’s (2004) measure of interstate parasite stress. White = analyses with the subsample of non-Hispanic Whites based on the rates of Chlamydia and Gonorrhea for non-Hispanic Whites. PRV = proportional reduction in variance.

Supporting parasite stress theory, as the F&T measure of parasite stress increased, respondents were more likely to think that the press should be restricted during wartime (Test 2) and that offensive speech should be censored (Test 3). Its effect in Test 4 (a replication on attitudes toward censoring offensive speech) was marginally significant (*p* = .065). These findings remained after we deleted the states that had fewer than 10 respondents.

We then repeated the above analyses by using C&G White as the Level-2 predictor variable to predict White respondents’ responses to the outcome variables. If parasite stress theory is correct, C&G White should also predict the outcome variables. However, as shown in Table 4, even though C&G White had a sizeable effect on wartime censorship attitudes (PRV = 23%), it did not reach the conventional threshold of statistical significance, *p* = .095. Further, there was no evidence that C&G White predicted attitudes toward censoring offensive speech (Tests 3 and 4). Parasite stress theory received support with the F&T measure of parasite stress but did not pass the more conservative test with C&G White.

### Tests of the Life-History Theory Explanation

Life history theory predicts that a slower LHS will be associated with stronger support for restrictions on press- and speech freedom. To test this prediction, we used Teen Birth Total and Teen Birth White in separate models to predict the outcome measures that were based on the full samples and the subsamples of non-Hispanic Whites. Gender and education level were covariates. Results are summarized in Table 5. Teen Birth Total significantly predicted wartime censorship attitudes (Test 2), but the effects were in the opposite direction to the prediction—individuals adopting a faster, instead of the predicted slower, LH strategy tended to think that the press should be restricted during wartime. There was no evidence that Teen Birth Total predicted attitudes toward censoring offensive speech (Test 3 and 4).

**Table 5:** Tests of life history theory.

		Press freedom in general (Test 1)		Wartime press freedom (Test 2)		Speech freedom (Test 3)		Speech freedom (Test 4)	
		State totals	White	State totals	White	State totals	White	State totals	White
Teenage birth	*b*			.12		.02	.04	.13	.14
	*t*(df)			3.67 (24.7)		< 1	1.35 (45.9)	1.42 (8.48)	1.81 (8.55)
	*p*			.001			.18	.19	.11
	Effect size			PRV = 61%			PRV = 14%	PRV = 15%	PRV = 29%
Education	*b*	-.06	-.07	-.14	-.17	-.15	-.14	-.45	-.44
	*t*(df)	-6.35 (1920)	-6.39 (1560)	-7.96 (990.0)	-8.03 (1560)	-10.6 (1973.3)	-9.02 (1605.5)	-16.4 (3137.9)	14.4 (2410.0)
	*p*	< .001	< .001	< .001	< .001	< .001	< .001	< .001	< .001
	Effect size	β = .14	.16	PRV = 43%	β = .28	PRV = 42%	37%	PRV = 16%	10%
Gender (male = 0)	*b*	.10	.08	.07	.02	.27	.25	.20	.26
	*t*(df)	3.24	2.57	1.19 (990.0)	< 1	6.12 (1975.5)	5.17 (1607.2)	2.94 (3136.7)	3.55 (2407.0)
	*p*	.001	.010	.24		< .001	< .001	.003	< .001
	Effect size	β = .07	.06	PRV = 8%		PRV = 20%	10%	PRV = 13%	14%

Note. PRV = proportional reduction in variance.

Unexpectedly, when Teen Birth White was used as the Level-2 predictor variable, the multilevel model failed to converge on the outcome measure for Test 2 (on wartime censorship attitudes) due to a non-positive definite Hessian matrix. This problem can be traced to a number of causes, including a lack of Level-2 variation, improper scaling of variables, and/or model misidentification [37]. Wartime censorship attitudes had significant between-state variation, χ^2^ = 4.06, *p* = .044-in fact, a similar model with C&G White as the Level-2 predictor variable was fitted (see above). We tried several remedies, including standardizing all variables and setting different starting values for model iteration, but the problem persisted. The fact that the model successfully converged with C&G White but not with Teen Birth White was replicated by using HLM [38], a program specialized in performing multilevel analyses. The effect of C&G White was thus not assessed in Test 2.

Test 3 and 4 on attitudes toward censoring offensive speech did not support life history theory. There was no evidence that Teen Birth White predicted the outcome measures. All findings remained after states with fewer than 10 states were deleted. Life history theory was not supported, but the large effect of Teen Birth Total (PRV = 61%) on wartime censorship attitudes is noteworthy.

### Competitive Tests

Because Tests 2 and 3 provided evidence for parasite stress and life history theories with full-sample analyses (see Tables 4 and 5), we compared effects of the F&T measure and Teen Birth Total by entering both of them into multilevel models with gender and education level as Level-1 covariates. Results are summarized in Table 6. For Test 2, Teen Birth Total emerged as a significant predictor but the F&T measure did not. But for Test 3, the F&T measure remained a significant predictor even after controlling for the effect of Teen Birth Total. In both tests, education level remained a significant Level-1 predictor variable.

**Table 6:** Competitive tests between parasite stress and life history theories.

		Wartime press freedom (Test 2)	Offensive speech (Test 4)
F&T	*b*	.02	.09
	*t*(df)	< 1	2.34 (65.7)
	*p*		.022
	PRV		50%
Teen birth	*b*	.11	-.02
	*t*(df)	2.83 (21.4)	< 1
	*p*	.010	
	PRV	48%	
Education	*b*	-.14	-.15
	*t*(df)	-7.94 (990)	-10.6 (1962.8)
	*p*	< .001	< .001
	PRV	42%	57%
Gender (male = 0)	*b*	.07	.27
	*t*(df)	1.19 (990)	6.19 (1977.2)
	*p*	.24	< .001
	PRV	8%	35%

### Effects of Individual Difference Variables

Across four tests and regardless of using the full samples or the subsamples of non-Hispanic Whites, respondents’ education level remained a robust and consistent predictor variable. Consistent with previous research [3], more educated respondents were less likely to think that press freedom should be restrained in general or during wartime (Tests 1 and 2) and were also less likely to think that offensive speech should be censored (Tests 3 and 4).

## Discussion

In this research, we compared three ecological explanations—climate-economic, parasite stress, and life history theories—for U.S. citizens’ attitudes toward press- and speech freedom.

### Implications for the Climato-Economic Explanation

Our analyses did not support climate-economic theory; the predicted interaction of climate demands and state wealth did not emerge in any of the four tests. Reflecting upon these null findings, one could argue that the U.S. as a whole should be regarded as a wealthy region as compared to, for instance, China, and it is thus not meaningful to divide the U.S. into wealthier versus poorer states. Indeed, Van de Vliert [39] found that higher climate demands were associated with stronger collectivism in China but were associated with weaker collectivism in the U.S. However, while climato-economic theory sets an objective reference point for measuring climate demands (i.e., 72°F), it does not do so for measuring regional wealth. Regional wealth is inherently comparative; while Mississippi may have a higher gross state product than many Chinese provinces, it is still less wealthy than other U.S. states.

Further, if climate-economic theory is correct, similar interaction effects between climate demands and regional wealth should be observed across different levels of cross-cultural comparisons. If the U.S. as a whole ought to be treated as a wealthy region, then the theory predicts that higher climate demands would be associated with less support for restricting freedom of expression. However, our analyses provided no evidence for a significant main effect of climate demands in any of the four tests.

State wealth emerged as a significant predictor variable in one test (Test 2 on wartime censorship attitudes). As can be expected, residents from wealthier states were more opposed to restricting press freedom even during wartime. This finding is consistent with previous findings that democratic attitudes and beliefs are more likely to develop and persist in more affluent regions [40,41]. However, a similar effect of state wealth was not observed in other tests in this research, and its main effect alone does not constitute evidence for climato-economic theory.

### Implications for Parasite Stress Theory

Parasite stress theory was supported in two tests when the F&T measure of parasite stress was used. These findings are largely consistent with Thornhill and Fincher’s [8] finding that higher parasite stress in a country is associated with higher degrees of institutional oppression of media freedom. Their study and ours suggest that, as regional parasite stress increases, not only will government place more restrictions on the free flow of information, but citizens are also likely to endorse those restrictions.

However, these findings should be treated with caution because the F&T measure is likely confounded with such LH variables as sexual strategy and extrinsic mortality rates [12]. Indeed, when only the subsamples of non-Hispanic White respondents were analyzed (thus controlling for extrinsic mortality rates), C&G rates, which constituted a significant portion of the F&T measure (*r* = .98), failed to predict any of the outcome measures in our tests. Nonetheless, it is also worth noting that the C&G rate measure provides a very conservative test of parasite stress theory. Both C&G are largely asymptomatic diseases (especially among women; [42,43]), and when they do show symptoms (e.g., burning while urinating), the symptoms are harder to detect than, for example, skin lesion. For parasite stress theory to work, however, it is necessary that people are able to detect symptoms (which may not reliably correlate with actual infection) in others so that the behavioral immune system is activated [13]. As such, STDs alone may not be enough to trigger behavioral immunity. Nevertheless, while these findings pose a further challenge to parasite stress theory when the theory is used to investigate cross-cultural differences in values and attitudes, they also call for more research to distinguish between parasite stress and LH variables [35].

### Implications for Life History Theory

Life history theory received no support. First, regardless of whether the full or sub-samples were used, there was no evidence that teen birth rates predicted attitudes toward censoring offensive speech. However, analyses did show a significant and strong effect of Teen Birth Total on wartime censorship attitudes. This finding is consistent with previous research [34] that found a positive relationship between teenage birth rates and endorsement of binding moral foundations, such as the valuation of ingroup loyalty. Rather than providing support for life history theory, these observations are consistent with the hypothesis that intergroup conflict heightens support for government and military independence and for checks on freedom [44].

Ours is not the first study that has shown effects of teenage birth rates that are opposite to the prediction of life history theory. For example, Hackman and Hruschka [12] predicted that states (e.g., the Southern states) that are characterized by faster LH strategies would be less religious, but found to the contrary that higher teenage birth rates were associated with higher levels of religiosity. Similarly, van Leeuwen et al. [35] predicted that faster LH strategies would be associated with weaker endorsement of binding moral foundations, but found that residents of the states with higher teenage birth rates more strongly endorsed binding moral foundations. While life history theory remains an important theory that has successfully explains many facets of human psychology and behavior [10,25,26], all of these findings—including our own—suggest that theoretical work is needed to better formulate of predictions based on life history theory when it is applied to explain cross-cultural differences in human values and psychology.

### Implications for the Individual Difference Approach

The ecological approach does not undermine the individual difference approach. In fact, our study showed a strong, consistent effect of education level on censorship attitudes. Future research should explore how ecological and individual difference variables combine to explain censorship attitudes (and, indeed, other outcomes of interest). In the context of our study, it is possible that individuals’ authoritarianism and conservatism [4,5] mediate the effect of regional parasite stress or LH variables on attitudes towards censorship. If validated, this approach would provide an integrative solution that reconciles the individual-difference approach and ecological explanations [45].

### Press Freedom and Speech Freedom

Our measures of interstate parasite stress and LHS predicted attitudes toward press freedom (during wartime) but not attitudes toward offensive speech. A possibility is that, while attitudes toward press- and speech freedom both concern the endorsement of free expression, the items concerning speech-freedom attitudes imply a greater level of interpersonal and social harm (e.g., allowing racists to have a public forum). If this is the case, higher teenage birth rates should predict stronger endorsement of restricting offensive speech, because faster LHS predicts weaker concerns with interpersonal harm [34]. However, we found no effects for teenage birth rates on attitudes toward restricting offensive speech. Future research is needed to further differentiate the attitudes toward restricting press freedom and speech freedom.

It is not uncommon to observe that right-wing press, which is more likely to be found in Southern states than in Northeastern states, intentionally publishes offensive content to intensify intergroup relations. This observation does not necessarily contradict our finding that citizens of Southern states, which are characterized with higher levels of parasite stress and faster life histories, showed stronger endorsement of restricting press freedom. The publication of offensive content often serves political goals, which are not necessarily consistent with those of common citizens. In fact, Southerners are believed to be generally more friendly and polite than people in other states (unless provoked [46]). None of our outcome measures contained phrases that cue to intergroup conflicts *within* the U.S., and we thus believe that our findings are in line with real life observations.

## Limitations

A limitation of our study is that the outcome measure of Test 1 (on whether the press has too much freedom in general) did not have sufficient between-state variations to perform multilevel analyses. This is likely because the outcome measure in that test was measured with a three-point scale, which is not ideal in generating variance. Another issue with the data is that the samples did not include enough African Americans to perform stratified analyses as we did with non-Hispanic Whites. It thus remains unknown whether the same findings that we observed with the full sample and the subsample of White respondents can be replicated with African Americans and other racial groups. While research based on secondary data is necessarily constrained by the quality of existing data, we would be excited to see other researchers test similar hypotheses with better-measured outcome variables and larger (and more representative) samples when they become available.

## Conclusions

Climato-economic, parasite stress, and life history theories all generate clear predictions regarding individual variations in attitudes toward freedom of expression. However, no one theory provided a successful explanation, despite some evidence for associations with measures of parasite stress and LH variables. Our tests of these theories demonstrate problems with possible misidentification of the underlying mechanisms (e.g., climato-economic theory, life history theory) and problematic measurement of key constructs (e.g., the F&T measure). Given the social importance of freedom of expression, future research should continue to explore the ultimate and proximate causes of cross-cultural and individual differences in the valuation of press, speech, and other forms of individual rights.

## Supporting Information

S1 Data Test 1; full sample and non-Hispanic White subsample. (XLSX)

S2 Data Test 2; full sample and non-Hispanic White subsample. (XLSX)

S3 Data Test 3; full sample and non-Hispanic White subsample. (XLSX)

S4 Data Test 4; full sample and non-Hispanic White subsample. (XLSX)

S1 File (Table A-E). Descriptive statistics, zero-order correlations, and supplementary tests.
(DOCX)
