# Supplementary material for: Predicting Attitudes toward Press- and Speech Freedom across the U.S.A.: A Test of Climato-Economic, Parasite Stress, and Life History Theories
Source: PLoS One. 2015 Jun 1;10(6):e0125241. doi: 10.1371/journal.pone.0125241 (PMC4451980; doi:10.1371/journal.pone.0125241)
Supplement: S1 File — Descriptive statistics, zero-order correlations, and supplementary tests. (DOCX) [file pone.0125241.s005.docx]

Supporting Information (S1)

**Table A. Descriptive Statistics of All Variables**

|  | *N* | Mean | Std. Deviation | Minimum | Maximum |
| --- | --- | --- | --- | --- | --- |
| The F&T Measure | 48 | -0.02 | 0.93 | -1.46 | 2.64 |
| C&G Total (1996-2011) | 48 | 197.98 | 74.69 | 70.21 | 410.39 |
| C&G White | 48 | 65.63 | 18.71 | 14.95 | 114.63 |
| Teen birth total | 48 | 39.58 | 11.88 | 18.00 | 62.00 |
| Teen birth white | 48 | 27.71 | 9.90 | 8.00 | 49.00 |
| Climate demands | 48 | 0.07 | 0.85 | -1.96 | 1.86 |
| State wealth | 48 | 0.00 | 0.92 | -1.71 | 2.32 |
| Attitudes toward press freedom in general (Test 1) | 1937 | 2.30 | 0.65 | 1.00 | 3.00 |
| Attitudes toward wartime press freedom (Test 2) | 999 | 2.52 | 0.93 | 1.00 | 4.00 |
| Attitudes toward speech freedom (Test 3) | 1993 | 2.51 | 1.02 | 1.00 | 4.00 |
| Attitudes toward speech freedom (Test 4) | 3146 | 7.23 | 1.96 | 1.00 | 12.00 |

*Note*. The F&T measure, or the measure of interstate parasite stress based on Fincher & Thornhill (2012), includes standardized scores. The mean is not zero because the standardization is based on all fifty states (including Alaska and Hawai‘i) but we only used the scores of the 48 contiguous states. The same applies to the climate-demands scores.

**Table B. Intercorrelations between State-Level Predictor Variables**

|  | F&T | C&G Total | C&G White | Teen birth total | Teen birth white | Climate demands | State wealth |
| --- | --- | --- | --- | --- | --- | --- | --- |
| F&T | 1 |  |  |  |  |  |  |
| C&G Total | .98*** | 1 |  |  |  |  |  |
| C&G White | -.09 | -.01 | 1 |  |  |  |  |
| Teen birth total | .59*** | .63*** | .45** | 1 |  |  |  |
| Teen birth white | .36* | .42** | -.48*** | .80*** | 1 |  |  |
| Climate demands | -.66*** | -.59*** | .10 | -.43** | -.28 | 1 |  |
| State wealth | -.16 | -.24 | -.47*** | -.54*** | -.78*** | .04 | 1 |

Note. *** *p* < .001 ** *p* < .01 * *p* < .05 (two-tailed)

**Table C. Zero-order Correlations of State- and Individual-Level Predictors with Outcome Measures for the Full-Sample Tests**

|  | Attitudes toward press freedom in general (Test 1) | Attitudes toward wartime press freedom (Test 2) | Attitudes toward offensive speech (Test 3) | | Attitudes toward offensive speech (Test 4) |
| --- | --- | --- | --- | --- | --- |
| *State-level* |  |  | |  |  |
| F&T | .42** (*N* = 48) | .35** (*N* = 48) | | .39** (*N* = 48) | .78* (*N* = 9) |
| C&G Total | .44** | .38** | | .44** | .76* |
| Teen birth total | .29* | .52*** | | .28 | .70* |
| Climate demands | -.28 | -.14 | | -.25 | -.58 |
| State wealth | -.24 | -.44 | | -.32* | -.57 |
| *Individual-level* |  |  | |  |  |
| Gender (male = 0) | .07** (*N* = 1937) | .04 (*N* = 999) | | .14*** (*N* = 1993) | .06 ** (*N* = 3146) |
| Education level | -.14*** (*N* = 1923) | -.25*** (*N* = 990) | | -.24*** (*N* = 1979) | -.29 *** (*N* = 3146) |

Note. *** *p* < .001 ** *p* < .01 * *p* < .05 (two-tailed)

**Table D. Zero-order Correlations of State- and Individual-Level Predictors with Outcome Measures for the Tests based on the Subsamples of Non-Hispanic Whites**

|  | Attitudes toward press freedom in general (Test 1) | Attitudes toward wartime press freedom (Test 2) | Attitudes toward offensive speech (Test 3) | | Attitudes toward offensive speech (Test 4) |
| --- | --- | --- | --- | --- | --- |
| *State-level* |  |  | |  |  |
| C&G White | .26 (*N* = 48) | .27 | | .09 | .22 (*N* = 9) |
| Teen birth white | .31* | .45** | | .29* | .78 * |
| Climate demands | -.25 | -.18 | | -.29* | -.59 |
| State wealth | -.19 | -.24 | | -.21 | -.66 |
| *Individual-level* |  |  | |  |  |
| Gender (male = 0) | .07** (*N* = 1566) | .03 (*N* = 791) | | .14*** (*N* = 1612) | .08 ** (*N* = 2416) |
| Education level | -.16*** (*N* = 1563) | -.28*** (*N* = 790) | | -.23*** (*N* = 1609) | -.29 *** (*N* = 2416) |

Note. *** *p* < .001 ** *p* < .01 * *p* < .05 (two-tailed)

**Table E. Tests of Parasite Stress Theory with C&G Total and C&G White**

|  |  | Press freedom in general  (Test 1) | | Wartime press freedom  (Test 2) | | Speech freedom  (Test 3) | | Speech freedom  (Test 4) | |
| --- | --- | --- | --- | --- | --- | --- | --- | --- | --- |
|  |  | State totals | White | State totals | White | State totals | White | State totals | White |
| C&G | *b* |  |  | .09  2.19 (57.1)  .033 | .06  1.71 (39.1)  .095 | .09  2.98 (87.6)  .004 | .003  < 1 | .16  2.10 (9.12)  .065 | -.01  < 1 |
|  | *t*(df) |  |  |  |  |  |  |  |  |
|  | *p* |  |  |  |  |  |  |  |  |
| Effect size | |  |  | PRV = 22% | PRV = 23% | PRV = 67% |  | PRV = 36% |  |
| Education | *b* | -.06  -6.29  < .001 | -.07  -6.41  < .001 | -.14  -7.86 (987.9)  < .001 | -.17  -8.29  < .001 | -.15  -10.6 (1959.7)  < .001 | -.14  -9.15 (1598.4)  < .001 | -.45  -16.4 (3142.0)  < .001 | -.44  -14.5 (2410.7)  < .001 |
|  | *t*(df) |  |  |  |  |  |  |  |  |
|  | *p* |  |  |  |  |  |  |  |  |
| Gender  (male = 0) | *b* | .10  3.28  .001 | .08  2.49  .013 | .07  1.16 (988.8)  .25 | .03  < 1 | .27  6.19 (1978.4)  < .001 | .25  5.20  (1607.1)  < .001 | .19  2.94 (3137.3)  .003 | .27  3.58 (2407.3)  < .001 |
|  | *t*(df) |  |  |  |  |  |  |  |  |
|  | *p* |  |  |  |  |  |  |  |  |

Notes. C&G rates = rates of Chlamydia and Gonorrhea from 1996 to 2011. State totals = non-stratified analyses. White = analyses with the subsample of non-Hispanic Whites. PRV = proportional reduction in variance.
